# 17-beta estradiol prevents cardiac myocyte hypertrophy by regulating mitochondrial E3 ubiquitin ligase 1

**DOI:** 10.1038/s41419-025-07389-3

**Published:** 2025-02-19

**Authors:** Ximena Calle, Valeria Garrido-Moreno, Brenda Becerra, Mayarling F. Troncoso, Juan Francisco Silva-Agüero, Emanuel Guajardo-Correa, Leslye Venegas-Zamora, Erik Lopez-Gallardo, Felipe Muñoz-Córdova, Fernanda Fredericksen, Sebastian Aedo-Cares, Allan Peñaloza-Otárola, Angelica Ortega, Angel Raya, Vinicius Maracaja-Coutinho, Mario Chiong, Valentina Parra, Sergio Lavandero

**Affiliations:** 1 Advanced Center for Chronic Diseases (ACCDiS), Facultad de Ciencias Químicas y Farmacéuticas & Facultad de Medicina, Universidad de Chile, Santiago, Chile; 2 Department of Internal Medicine (Cardiology Division), University of Texas Southwestern Medical Center, Dallas, Texas USA; 3 Millennium Institute Center for Genome Regulation, Santiago, Chile; 4 Regenerative Medicine Program, Institut d’Investigació Biomèdica de Bellvitge—IDIBELL, Program for Clinical Translation of Regenerative Medicine in Catalonia—P-[CMRC], and Center for Networked Biomedical Research on Bioengineering, Biomaterials and Nanomedicine (CIBER-BBN), L’Hospitalet de Llobregat, Barcelona, Spain; 5 Institució Catalana de Recerca i Estudis Avançats (ICREA), Barcelona, Spain; 6 SYSTEMIX Center for Systems Biology, O’Higgins University, Rancagua, Chile

**Keywords:** Heart failure, Mechanisms of disease

## Abstract

Cardiac hypertrophy is a cellular process characterized by the increased size of cardiomyocytes in response to a high workload or stress. 17-beta estradiol (E2) has cardioprotective and anti-hypertrophic effects by maintaining mitochondrial network and function. MUL1 is a mitochondrial ubiquitin ligase directly involved in the control of mitochondrial fission and mitophagy. Studies from our group and others have previously shown that cardiomyocyte hypertrophy is associated with mitochondrial fission and dysfunction. These findings led us to study in vitro whether E2 regulates MUL1 to prevent cardiac hypertrophy, mitochondrial fission, and dysfunction induced by the catecholamine norepinephrine (NE). Our results showed that NE induces hypertrophy in cultured rat cardiomyocytes. Pre-treatment with E2 (10-100 nM) prevented the NE-dependent increases in cell perimeter and the hypertrophic stress markers ANP and BNP at both the protein and mRNA levels. NE induced the fragmentation of the mitochondrial network and reduced ATP levels, effects that were both prevented by E2. *In* s*ilico* analysis suggested a putative binding site for estrogen receptors on the MUL1 gene promoter. In accordance with this finding, E2 prevented increases in MUL1 mRNA and protein levels induced by NE. Our data also showed that a siRNA MUL1 knockdown counteracted NE-induced cardiomyocyte hypertrophy and mitochondrial dysfunction, mirroring the protective effect triggered by E2. In contrast, a MUL1 adenovirus did not prevent the E2 protection from cardiomyocyte hypertrophy. Further, in vivo analysis in a transgenic mouse model overexpressing MUL1 revealed that only young male mice overexpressed the protein. Consequently, they exhibited increased levels of the hypertrophic marker ANP, an elevated heart weight, and larger cardiomyocyte size. Therefore, our data demonstrate that 17-beta estradiol prevents cardiac myocyte hypertrophy by regulating MUL1.

## Introduction

Heart failure is a pathological condition in which the heart cannot meet the metabolic demands of the body [1]. It often results from hypertension, valve disease, myocardial infarction, or inherited disorders, making it a terminal feature of cardiovascular diseases [1]. Despite advances in treatment, heart failure remains a leading cause of hospitalizations and healthcare expenditures, with persistently high morbidity and mortality rates [2, 3]. This underscores the urgent need to uncover molecular and cellular mechanisms driving heart failure to improve therapeutic strategies.

Cardiac hypertrophy is an early adaptive response to stress where cardiomyocytes enlarge and remodel to enhance contractile capacity [1]. While initially compensatory, sustained hypertrophy promotes ventricular dilation and progression to heart failure [4–6]. Hormonal differences influence hypertrophy susceptibility; premenopausal women experience reduced hypertrophy risk compared to men due to higher estrogen levels [7, 8]. This protection diminishes post-menopause, correlating with reduced estrogen and increased androgen levels [7, 9, 10]. Notably, hormone replacement therapy using estrogens benefits left ventricular hypertrophy independently of blood pressure or insulin resistance modulation [8, 11, 12].

The main circulating estrogen, 17-β-estradiol (E2), protects the cardiovascular system through diverse mechanisms, including mitochondrial preservation. E2 enhances oxidative phosphorylation, biogenesis, mitochondrial structure, antioxidant responses, and cell survival while maintaining mitochondrial dynamics [13–17]. These dynamics—encompassing biogenesis, fusion, fission, and degradation—are essential for homeostasis, and their dysregulation is linked to cardiac disease [18, 19].

Our previous studies demonstrated that norepinephrine (NE) induces cardiomyocyte hypertrophy through mitochondrial fission. This process is mediated by the Ca^2+^-calcineurin pathway, which activates dynamin-related protein-1 (DRP1), leading to fragmented mitochondria and impaired function [18]. These effects can be countered by dominant-negative DRP1 or angiotensin (1-9) treatment, highlighting the importance of mitochondrial dynamics in hypertrophy progression [18, 20].

Mitochondrial E3 ubiquitin ligase 1 (MUL1), located in the outer mitochondrial membrane, regulates mitochondrial dynamics through DRP1 SUMOylation and mitofusin-2 (MFN2) ubiquitination [21, 22]. MUL1 is upregulated in several models of cardiac hypertrophy, promoting mitochondrial fragmentation and dysfunction [22–26]. For instance, phenylephrine-induced hypertrophy in rat cardiomyocytes increases MUL1 levels while decreasing MFN2 in a MUL1-dependent manner [27]. MUL1 also mediates lipotoxicity-induced mitochondrial fission in hypertrophic models [28], underscoring its role in cardiac disease mechanisms.

Based on this evidence, we investigated the involvement of MUL1 in NE-induced cardiomyocyte hypertrophy and its modulation by E2. Our results reveal that MUL1 upregulation drives mitochondrial fragmentation and hypertrophy, processes prevented by E2 treatment. E2 downregulates MUL1, restoring mitochondrial dynamics and uncovering a novel cardioprotective mechanism through mitochondrial regulation.

## Results

### E2 protects against norepinephrine-induced cardiomyocyte hypertrophy

**Fig. 1:**
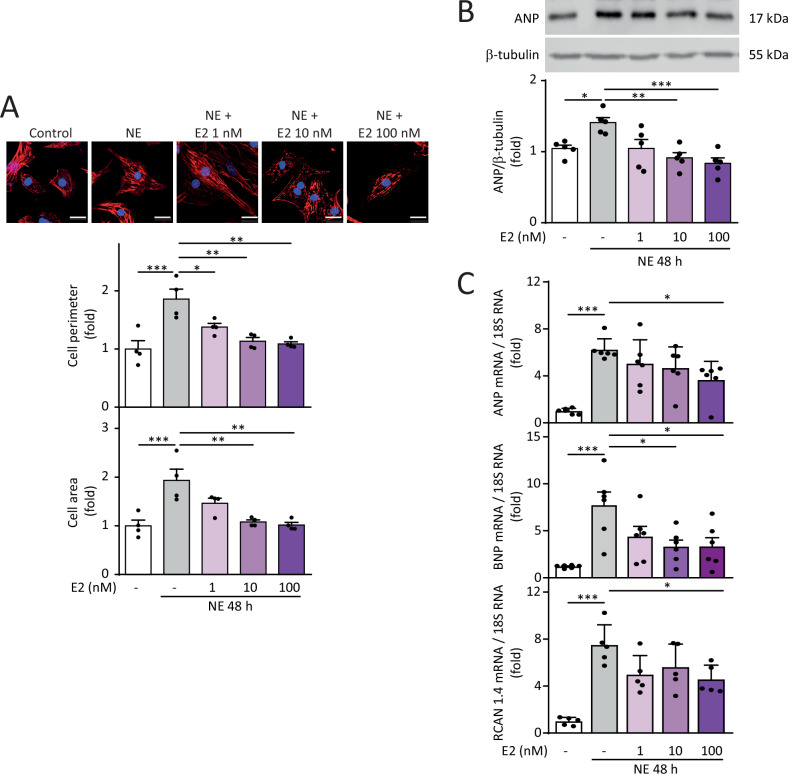
Effect of E2 on NE-induced cardiomyocyte hypertrophy. **A** Primary cultured NRVMs were treated with E2 (0, 1, 10, and 100 nM) for 6 h and then with NE (20 μM) for 48 h. NRVMs were stained with rhodamine-phalloidin. Nuclei were stained with DAPI. Images were captured using confocal microscopy (upper panel). Scale bar = 20 μm. Cell perimeter and cell area were determined (lower panel) (*N* = 4). **B** Determination of ANP levels using western blotting (*N* = 5). **C** ANP (*N* = 6), BNP (*N* = 6), and RCAN 1.4 (*N* = 5) mRNA levels were determined by RT-qPCR. 18S RNA was used to normalize the data. Values correspond to the mean ± SEM. Each independent experiment is displayed as a dot in the graphs. The data were analyzed using one-way ANOVA and Tukey’s post-test for multiple comparisons. *p < 0.05, **p < 0.01, and ***p < 0.001.

NE induced hypertrophy in NRVMs, as determined by increased cell area and perimeter assessed by confocal microscopy and cytoskeletal staining using rhodamine phalloidin. Pre-treatment with E2 at concentrations ranging from 1 to 100 nM prevented the increase in NE-dependent cell perimeter at 48 h of induction. However, only pre-treatment with 10 nM and 100 nM E2 prevented the increase in cell area induced by NE for 48 h (Fig.1A). Subsequently, we analyzed whether E2 prevented the increase in the hypertrophic stress markers ANP and BNP at both protein and mRNA levels. Concentrations of 10 and 100 nM E2 prevented the increase in ANP protein levels in hypertrophic NRVMs (Fig. 1B). Concerning mRNA levels, 100 nM E2 prevented the hypertrophy-inducing effects of NE on increased ANP, BNP, and RCAN 1.4 levels (Fig. 1C), this last one being a readout of calcineurin activity [29]. Changes in these markers were not observed in NRVMs treated only with E2 (Suppl Fig. 1). Therefore, E2 prevents NE-induced hypertrophy in NRVMs.

### E2 prevents mitochondrial dysfunction in cultured cardiomyocytes treated with NE

**Fig. 2:**
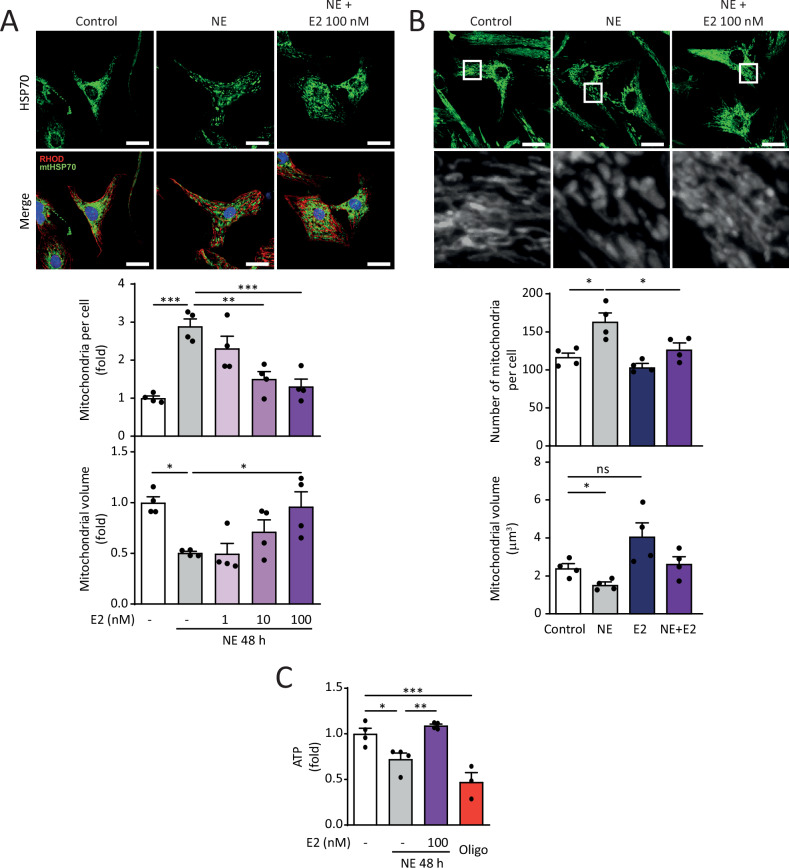
Effect of E2 on mitochondrial morphology and function in NE-treated cardiomyocytes. Primary cultured NRVM were treated with E2 (0, 1, 10, 100 nM) for 6 h and then with NE (20 μM) for 48 h. **A** Mitochondria were visualized by mtHsp70 immunolabeling (green) (*N* = 4). Cell contours were delimited by rhodamine-phalloidin staining (red) and nuclei with DAPI (blue). Scale bar = 20 μm. **B** NRVMs were stained with MTG (400 nM). Images were obtained by confocal microscopy and analyzed to determine the number of mitochondria per cell and the relative mitochondrial volume (*N* = 4). Scale bar = 20 μm. **C** Intracellular ATP content was determined by luciferin-luciferase assay. Oligomycin (oligo, 200 nM) was used as a control (*N* = 4). The data correspond to the mean ± SEM. Each independent experiment is displayed as a dot in the graphs. Results were analyzed using one-way ANOVA followed by multiple Tukey’s comparisons. *p < 0.05, **p < 0.01, and ***p < 0.001.

Bioenergetic dysfunction and disruption in mitochondrial dynamics are two parameters directly involved in the development of cardiovascular diseases and cardiac hypertrophy [30]. We evaluated the mitochondrial dynamics of NRVMs treated with NE and E2 in fixed (Fig. 2A) and live NRVMs (Fig. 2B). In both experiments, NE induced the fragmentation of the mitochondrial network, as indicated by an increased mitochondrial number and decreased relative volume. Pre-treatment with 100 nM E2 preserved the integrity of the mitochondrial network, which protects against NE-induced hypertrophy, as shown by mitochondria that maintained their larger volume and lower number (Fig. 2A, B). Concerning mitochondrial bioenergetics, NE significantly reduced ATP levels in NRVMs, as we have previously reported [18]. Consistently, pre-treatment with 100 nM E2 prevented this effect (Fig. 2C). Furthermore, treatment with E2 alone did not modify mitochondrial parameters in NRVMs (Suppl. Fig. 2).

These data suggest that E2 protects against NE-induced hypertrophy while also preserving the mitochondrial network and ATP synthesis. These data agree with findings reported in the literature for several cardiovascular disease models [16].

### MUL1 induction during NRVM hypertrophy is prevented by E2

**Fig. 3:**
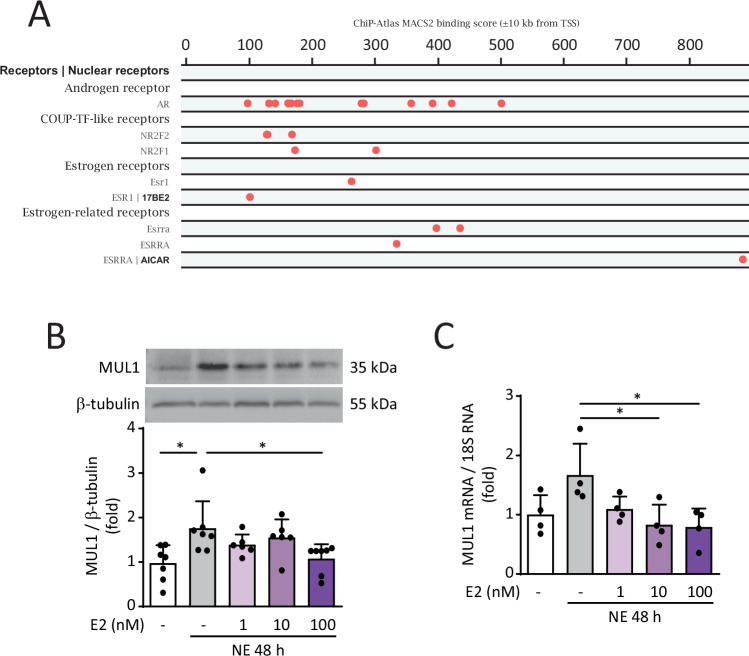
Effect of NE on MUL1 in cultured cardiomyocytes. **A** Chip-atlas data representing the MACS2 pic values generated within 10 kb of the MUL1 gene transcription start site. Each dot represents a putative binding site. Primary cultured NRVMs were treated with E2 (0, 1, 10, 100 nM) for 6 h and then with NE (20 μM) for 48 h. **B** Protein extracts were obtained and analyzed by western blotting using a MUL1 antibody (*N* = 7). β-tubulin was used as a loading control. **C** MUL1 mRNA levels were determined by RT-qPCR (*N* = 4). 18S RNA was used to normalize the data. The data correspond to the mean ± SEM. Each independent experiment is displayed as a dot in the graphs. Results were analyzed using one-way ANOVA followed by multiple Tukey’s comparisons. *p < 0.05.

Since MUL1 is an important protein in the induction of cardiac hypertrophy [27], we aimed to assess whether E2 prevented its induction. Moreover, studies have described that MUL1 controls mitochondrial fission by regulating the ubiquitination of the fusion protein MFN2, as well as the SUMOylation and stabilization of the fission protein DRP1 [28]. To explore potential regulatory mechanisms, we performed an in silico analysis using data from the ChIP Atlas and the Signaling Pathway Project to identify likely regulatory interaction sites in the MUL1 promoter based on ChIP-Seq datasets. Interestingly, these datasets revealed that sexual steroid hormone receptors, including androgen receptors (AR), estrogen receptor α (ERα), and estrogen-related receptors (ERRs), may bind to regulatory regions of the MUL1 promoter (Fig. 3A). While AR is not directly regulated by E2, these findings raise the possibility that E2 could influence MUL1 mRNA levels via interactions with ERα or ERRs. This hypothesis aligns with our results, which show that E2 prevents MUL1 induction in response to hypertrophic stimuli, suggesting that the cardioprotective effects of E2 may involve the modulation of MUL1 expression.

We determined that NE treatment increased MUL1 protein and mRNA levels (Fig. 3B, C). Interestingly, 100 nM E2 prevented this rise in MUL1 levels in NRVMs treated with NE (Fig. 3B, C). However, neither NE or E2 treatments altered the protein levels of MFN2, which is a target of MUL1 (Suppl. Fig. 3A, B). Therefore, to figure out how NE induced mitochondrial network fragmentation, we evaluated the activating phosphorylation of DRP1 at Ser616. The treatment of NRVMs with NE increased DRP1 phosphorylation. This effect was prevented by 100 nM E2 (Suppl. Fig. 3C). E2 alone did not modify DRP1 phosphorylation (Suppl. Fig. 3D). These results suggest that E2 prevents mitochondrial network fragmentation through the regulation of DRP1 phosphorylation.

### MUL1 siRNA mimics the protective effect of E2 on NRVMs

**Fig. 4:**
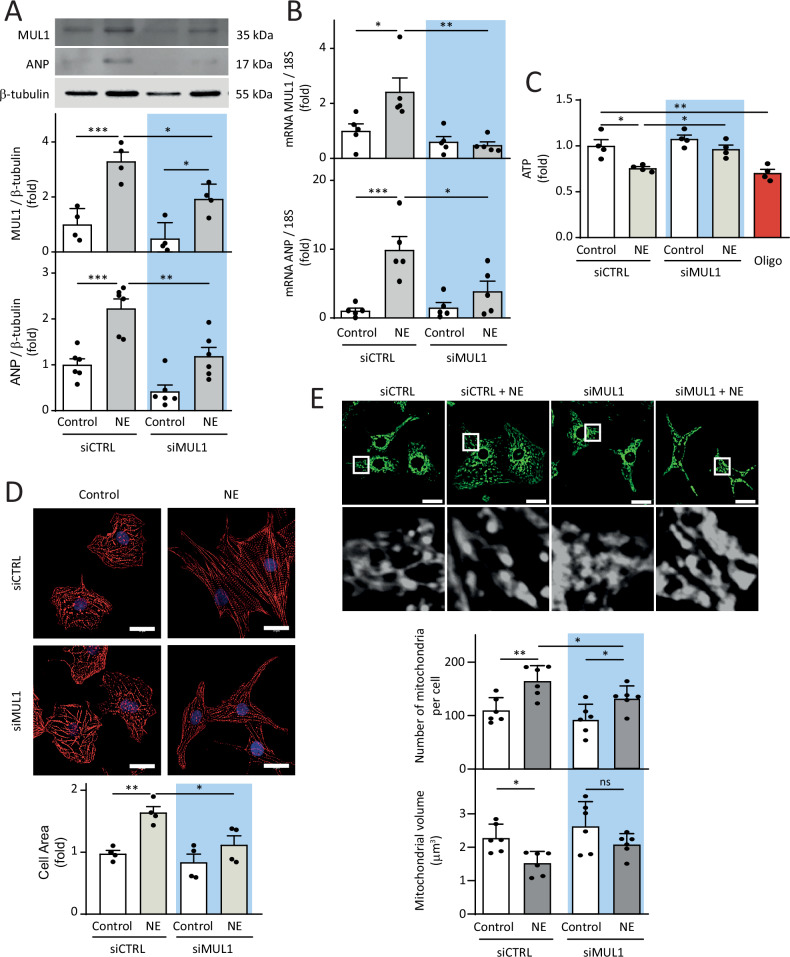
Role of MUL1 in NE-induced cardiomyocyte hypertrophy. Cardiomyocytes were treated with a MUL1 siRNA (siMUL1) or a scrambled siRNA (siCTRL) for 24 h and then treated with NE (20 μM) for 48 h. **A** Protein extracts were obtained, and MUL1 (*N* = 4) and ANP (*N* = 6) protein levels were determined by western blotting. β-tubulin was used as a loading control. **B** MUL1 (*N* = 5) and ANP (*N* = 5) mRNA levels were determined by RT-qPCR. 18S RNA was used to normalize the data. **C** Intracellular ATP levels were assessed by a luciferin-luciferase assay (*N* = 4). Oligomycin (Oligo, 200 nM) was used as a control. **D** NRVMs were stained with rhodamine-phalloidin (red) and nuclei with DAPI (blue). Confocal images were obtained, and cell area was determined (*N* = 4). Scale bar = 20 μm. **E** NRVMs were stained with MTG (400 nM). Images were obtained by confocal microscopy and analyzed to determine the number of mitochondria per cell and the relative mitochondrial volume (*N* = 6). Scale bar = 20 μm. The values correspond to the mean ± SEM. Each independent experiment is displayed as a dot in the graphs. Results were analyzed using two-way ANOVA followed by multiple Tukey’s comparisons. *p < 0.05, **p < 0.01, and ***p < 0.001.

Since we anticipated the potential application of MUL1 as a pharmacological target in hypertrophy, we investigated whether silencing MUL1 using siRNA (siMUL1) mimicked the anti-hypertrophic effect of E2. We confirmed that siMUL1 effectively reduced MUL1 protein levels compared to siCTRL (Fig. 4A). Then, we found that siMUL1 significantly prevented the increase in ANP protein levels induced by NE in NRVMs compared to siCTRL (Fig. 4A). The corresponding mRNA levels confirmed both effects (Fig. 4B). This information directly correlated with the reduction of cell area induced by siMUL1 in NRVMs treated with NE (Fig. 4D). As described above, NE-induced hypertrophy decreases ATP levels, which is associated with mitochondrial dysfunction. In this regard, MUL1 silencing prevented the decrease in ATP levels in NRVMs treated with NE for 48 h (Fig. 4C) and mitigated the mitochondrial fragmentation triggered by NE. This effect was evidenced by a significant reduction in the number of mitochondria per cell and a less pronounced decrease in mitochondrial volume when comparing the siCTRL and siMUL1 conditions after NE treatment (Fig. 4E). These findings suggest that MUL1 knockdown, similar to E2, exerts a protective effect against NE-induced hypertrophy and mitochondrial dysfunction in NRVMs.

### MUL1 overexpression does not reverse the protective effect of E2 on NRVMs

**Fig. 5:**
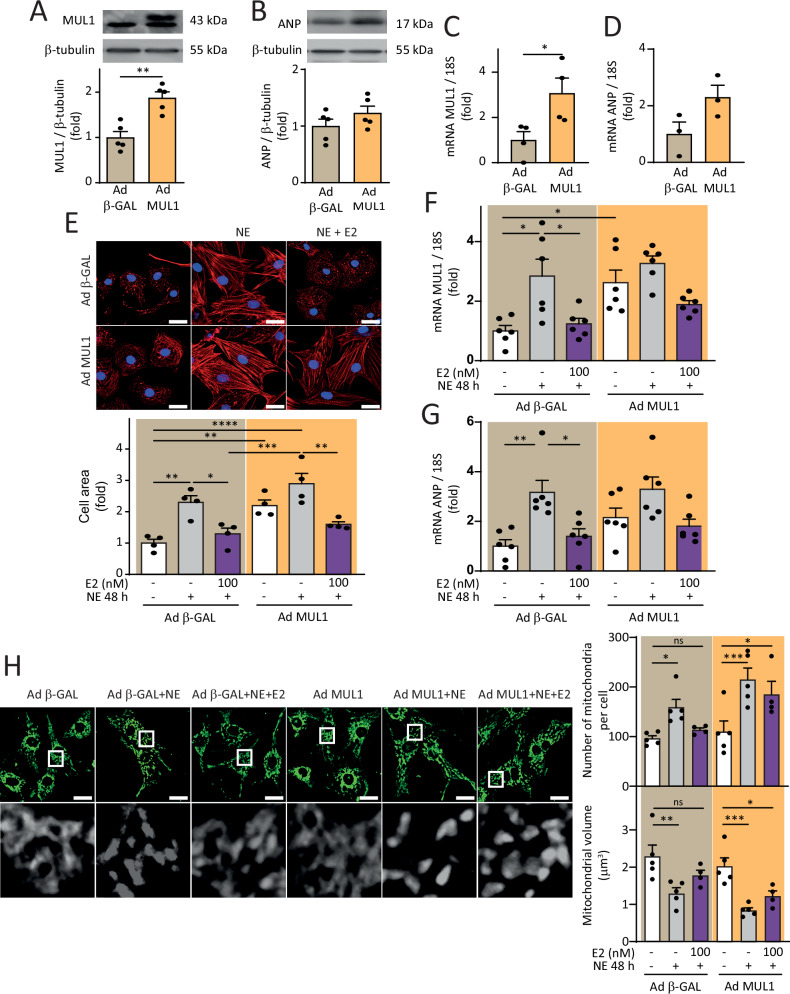
Role of MUL1 on the anti-hypertrophic effects of E2 in NE-treated cardiomyocytes. NRVMs were treated with Ad MUL1 or Ad β-GAL (control) with a MOI = 100 and incubated for 24 h. **A** MUL1 (*N* = 5) and **B** ANP (*N* = 5) protein levels were determined by western blotting. β-tubulin was used as a loading control. **C** MUL1 (*N* = 4) and **D** ANP (*N* = 3) mRNA levels were assessed by RT-qPCR. 18S RNA was used to normalize the data. The data correspond to the mean ± SEM. Results were analyzed using a one-way ANOVA test followed by multiple Tukey’s comparisons. *p < 0.05, **p < 0.01. **E** NRVMs were stained with rhodamine-phalloidin (red) and nuclei with DAPI (blue). Confocal images were obtained, and cell area was determined (*N* = 4). **F** MUL1 (*N* = 6) and **G** ANP (*N* = 6) mRNA levels were determined by RT-qPCR. 18S RNA was used to normalize the data. **H** NRVMs were stained with MTG (400 nM). Images were obtained by confocal microscopy and analyzed to determine the number of mitochondria per cell and the relative mitochondrial volume (*N* = 5). Scale bar = 20 μm. The values correspond to the mean ± SEM. Each independent experiment is displayed as a dot in the graphs. Results were analyzed using 2-way ANOVA followed by multiple Tukey’s comparisons. *p < 0.05, **p < 0.01, ***p < 0.001, and ****p < 0.0001.

Next, we evaluated whether overexpressing MUL1 using an Ad MUL1 (human) construct could block the protective effect of E2 on NE-induced hypertrophy in NRVMs. We confirmed that Ad MUL1 effectively increased MUL1 protein and mRNA levels (Fig. 5A, C) while not significantly increasing ANP protein and mRNA levels (Fig. 5B, D). As described above, pre-treatment with E2 prevented the rise in MUL1 and ANP mRNA levels induced by NE (Fig. 1C and Fig. 3B). Therefore, it was relevant to evaluate whether Ad MUL1 influenced these parameters. NE treatment did not modify the basal increase in cell area due to MUL1 overexpression (Fig. 5E), and the pre-treatment with E2 prevented NE-dependent cell area increases in both the control Ad β-GAL and the Ad MUL1-treated NRVMs (Fig. 5E). Interestingly, NE treatment did not further increase MUL1 mRNA levels in Ad MUL1-treated NRVMs compared to control Ad β-GAL-treated NRVMs (Fig. 5F), while the pre-treatment with E2 failed to significantly decrease MUL1 mRNA levels in Ad MUL1-treated NRVMs (Fig. 5F). Similarly, NE treatment did not further increase ANP mRNA levels in Ad MUL1-treated NRVMs compared to control Ad β-GAL-treated NRVMs (Fig. 5G), and the pre-treatment with E2 did not significantly decrease ANP mRNA levels in Ad MUL1-treated NRVMs (Fig. 5G). Further, regarding mitochondrial morphology, Ad MUL1-treated NRVMs were resistant to the protection exerted by E2 against mitochondrial fragmentation, as assessed by the quantification of the number of mitochondria per cell and the evaluation of the individual mitochondrial volume. Interestingly, the sole overexpression of MUL1 in NRVMs had no effects on mitochondrial fragmentation, but it seemed to enhance the effects of NE, although without statistical significance at 48 h (Fig. 5H). Based on these results, a compensatory mechanism might maintain restricted levels of MUL1 mRNA, even under pathological stimuli combined with overexpression, thus limiting the effects to the mitochondria but not affecting all aspects of the hypertrophy phenotype. These results reaffirm that MUL1 is a potent pharmacological target to emulate the cardioprotective effect of E2 and suggest that it could potentially avoid the side effects associated with this steroid hormone [31].

### Descriptive analysis of MUL1 in a human hypertrophic cell model and functional insights from in vivo studies using a transgenic MUL1 mouse model

**Fig. 6:**
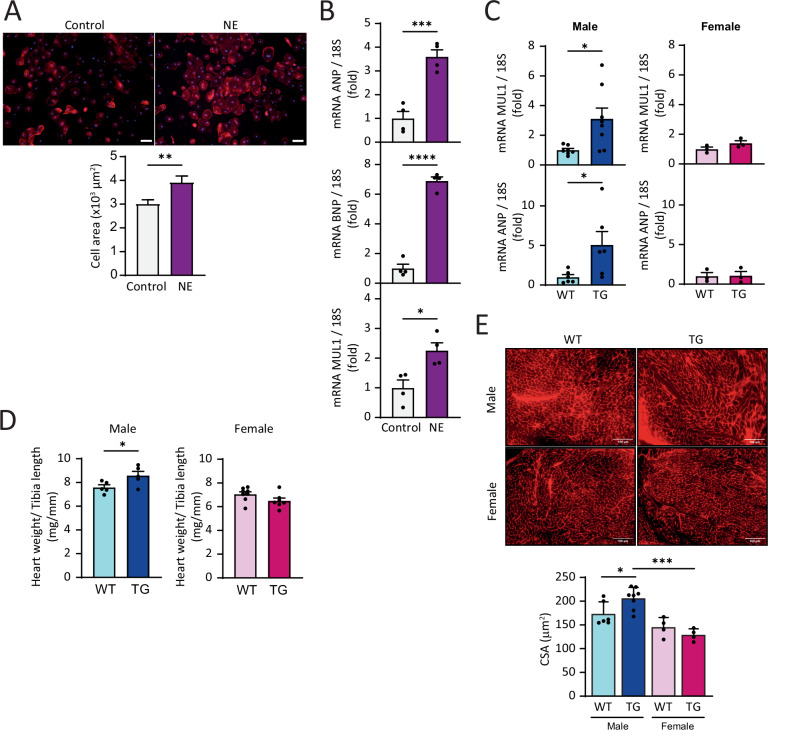
MUL1 evaluation in a human model of cardiac hypertrophy and functional insights in an in vivo transgenic mice MUL1 model. **A** CM-hiPSC matured for 30 days were treated with NE (10 μM) for 10 days. CM-hiPSC were stained with rhodamine-phalloidin. Nuclei were stained with DAPI. Images were captured using confocal microscopy (upper panel). Scale bar = 50 μm. Cell area was determined (lower panel) (*N* = 4). **B** ANP (*N* = 4), BNP (*N* = 4), and MUL1 (*N* = 4) mRNA levels were determined by RT-qPCR. 18S RNA was used to normalize the data. The values correspond to the mean ± SEM. Each independent experiment is displayed as a dot in the graphs. Results were analyzed using a student *T*-test. *p < 0.05, **p < 0.01, ***p < 0.001, and ****p < 0.0001. Cardiac hypertrophic markers were evaluated in a transgenic mouse model overexpressing MUL1 (TG) and their respective wild-type controls (WT). **C** Expression levels of MUL1 mRNA and the hypertrophic marker ANP were measured in male and female mice at 37 weeks of age (MUL1: *N* = 7 WT male; *N* = 8 TG Male, *N* = 3 WT female, *N* = 3 TG female; ANP: *N* = 6 WT male; *N* = 6 TG Male, *N* = 3 WT female, *N* = 3 TG female). **D** Heart weight-to-tibia length ratio evaluation in male and female WT and TG mice (*N* = 5 WT male, *N* = 5 TG male, *N* = 8 WT female, *N* = 7 TG female). **E** Cardiomyocyte cross-sectional area (CSA) analysis in WT and TG male and female 37 weeks mice (*N* = 6 WT male, *N* = 8 TG male, *N* = 4 WT female, *N* = 4 TG female). Values correspond to the mean ± SEM. Each animal is displayed as a dot in the graphs. The data were analyzed using a Mann-Whitney nonparametric test. *p < 0.05, and ***p < 0.001.

To determine whether the increase in MUL1 in response to a hypertrophic stimulus like NE was exclusive to NRVM primary cultures, we assessed MUL1 RNA levels in a human cardiomyocyte culture derived from human induced pluripotent stem cells (hiPSCs). iPSCs were differentiated into cardiomyocytes (CM-hiPSCs) following the protocol described by Lian et al. [32] and allowed to mature for 30 days. On day 30, 10 µM NE was added to the medium and maintained for 10 days, with medium changes every 48 h. At the end of this period, NE-induced hypertrophy in CM-hiPSCs was confirmed by an increased cell area, as evaluated by fluorescence microscopy and cytoskeletal staining using rhodamine phalloidin (Fig. 6A). Subsequently, we analyzed whether NE treatment also increased the expression of hypertrophic stress markers ANP and BNP, as well as MUL1, at the mRNA level. A significant increase was observed for all markers following NE treatment (Fig. 6B). These data suggest that human CM-hiPSCs also exhibit increased MUL1 expression after hypertrophic induction with NE.

Finally, to provide a more physiological context to our analysis, we measured various cardiac hypertrophic markers in a transgenic mouse model overexpressing MUL1. Given the importance of estrogens in the onset of cardiac disease and their role in MUL1 regulation, as described in our previous results, we conducted these measurements in male and female mice at 37 weeks of age. As shown in Fig. 6C, only male transgenic MUL1 mice exhibited increased levels of MUL1 mRNA, along with the hypertrophic marker ANP, consistent with our findings using Ad MUL1 (Fig. 5F), where restricted levels of MUL1 mRNA appeared particularly prominent in females. Furthermore, only male mice developed cardiac hypertrophy, as assessed by the heart weight-to-tibia length ratio, supporting the protective role of E2 in the hypertrophic response associated with increased MUL1 expression (Fig. 6D). Similarly, analysis of the cardiomyocyte cross-sectional area (CSA) revealed that only male mice exhibited enlarged cardiomyocytes, while female cardiomyocytes remained smaller in both WT and transgenic conditions (Fig. 6E). Taken together, these results suggest that MUL1 overexpression is associated with a hypertrophic cardiac response, but that the presence of E2 mitigates this response.

## Discussion

Our work is the first to report the relationship between the anti-hypertrophic effect of E2 and the regulation of MUL1 protein levels in cultured rat cardiomyocytes and a transgenic mouse model overexpressing MUL1. Additionally, using an in silico approach, we propose a putative regulation of MUL1 by the estrogen receptor.

The modulation of E2 and the ER has been extensively studied due to their direct impact on cardiomyocytes, exhibiting metabolic and antihypertrophic effects. Our study corroborated that E2 prevents the hypertrophy of cardiomyocytes induced by NE. Several mechanisms and signaling pathways have been proposed to explain this E2 action, such as calcineurin-NFAT [33], PI3K-AKT-mTOR [34], ERK1/2, PDK1-AKT [35], and KLF5 [36], among others. Our work adds a new possible target for downstream E2 activity, the protein MUL1.

MUL1, a mitochondrial ubiquitin ligase, plays a crucial role in regulating the levels, locations, and functions of various proteins, thus contributing to diverse cellular processes [22, 28]. Prior research has shown that MUL1 levels increase during cardiomyocyte hypertrophy induced by phenylephrine [27], a synthetic α1-adrenergic agonist that replicates the effects of endogenous catecholamines like NE, as well as by saturated fatty acids like myristic acid [30]. Furthermore, other studies have shown that inhibiting MUL1 effectively prevents cardiac hypertrophy in these previously described experimental contexts [26, 33]. Building upon this knowledge, our work confirms that reducing MUL1 expression prevents NE-induced cardiomyocyte hypertrophy. This finding suggests that targeting MUL1 levels could represent a promising biomedical strategy for preventing cardiomyocyte hypertrophy.

As previously mentioned, our study also showed that E2 prevented the increase in MUL1 levels induced by NE. However, the relationship between E2 and MUL1 remains unclear. E2 exerts its biological activity through three receptors: GPER1, situated in the endoplasmic reticulum and cytoplasmic membrane, whose activation initiates the rapid non-genomic activity, and the canonical receptors ERα and ERβ. Both ERs are intracellular receptors that, upon activation, translocate to the nucleus and bind to specific DNA sequences known as estrogen response elements (EREs), thereby regulating gene expression [16, 37]. Our previous research identified the transcription factor FoxO1 as a potential positive regulator of MUL1 expression [38]. However, since the regulation of MUL1 expression is not fully understood yet, we aimed to investigate the presence of EREs within the MUL1 promoter sequence using computational methods. Intriguingly, our analysis revealed the presence of hormone regulatory sites within the MUL1 DNA sequence, including those for the ER, ERR, and AR. Therefore, we hypothesize that ER may exert a negative regulatory influence on MUL1 expression. While these findings shed light on MUL1 regulation, experimental validation is still necessary to confirm these speculations, which present a promising avenue for future research.

MUL1 also regulates the levels of proteins involved in mitochondrial dynamics, a process that governs mitochondrial morphology, abundance, and function. Under physiological conditions, the interplay between mitochondrial fusion and fission balances this process [19, 30], which is regulated by various proteins that facilitate mitochondrial fusion and fission, including MFN2 and DRP1. MUL1 ubiquitinates MFN2, a protein involved in mitochondrial fusion, targeting it for degradation, while it SUMOylates DRP1, a protein involved in mitochondrial fission, promoting its stabilization within the mitochondria. These events collectively shift the balance towards mitochondrial fission, a process that our group [18, 20] and others [39, 40] have associated with the hypertrophic phenotype in cardiomyocytes characterized by reduced oxidative phosphorylation and ATP synthesis. Previous studies have illustrated that MUL1 promotes mitochondrial fragmentation in hypertrophic cardiomyocytes [27, 38]. Similarly, Cheng et al. showed that bisphenol B (BPF), an ERβ antagonist, activates calcineurin in cardiomyocytes, leading to the dephosphorylation of DRP1 at Ser637, thus promoting mitochondrial fission and reducing ATP levels [37]. In the present study, we showed that E2 prevents both mitochondrial fission and the reduction of ATP levels induced by NE, suggesting that E2 or ER could deactivate DRP1. Consistently, we depicted that E2 prevents the NE-induced phosphorylation of DRP1 at Ser616, a post-translational modification that, unlike Ser637 phosphorylation, translocates DRP1 to the mitochondria, promoting its fission. Therefore, DRP1 is a common factor between MUL1 and E2. It is yet to be ascertained whether the observed changes in DRP1 Ser616 are due to modifications in total protein levels resulting from its stabilization and whether E2 can also regulate phosphorylation at Ser637. Furthermore, although we investigated the involvement of MFN2, we did not observe any changes in its levels induced by E2. Nonetheless, it would be intriguing for future studies to explore the possibility of a reciprocal and opposing relationship between E2-MUL1 and DRP1.

Cardiac hypertrophy precedes functional damage, leading to heart failure [4–6], making the study of its development and underlying mechanisms paramount. Notably, evidence suggests that cardiovascular risk is lower in women compared to men before menopause, a period characterized by a drastic decline in estrogen levels that coincides with increased cardiovascular risk in women [8, 11, 12]. Consequently, numerous studies have explored the cardioprotective effects of estrogens and hormone replacement therapy, yielding conflicting results [41, 42]. Thus, it is imperative to elucidate the mechanisms underlying the cardioprotective effects of estrogen, particularly focusing on potential downstream therapeutic targets. Our research sheds light on the role of MUL1 in regulating cardiac hypertrophy and its potential implications for heart failure development. Is MUL1 expression increased in postmenopausal women or aged animal models? Do MUL1 levels rise with age? Answering these questions could provide valuable insights into evaluating cardiac health during aging and understanding why postmenopausal women lose their cardiac health advantage. While it remains unclear whether estrogen directly influences MUL1 or if both act through a shared pathway to prevent hypertrophy, our findings suggest that targeting MUL1 could replicate the antihypertrophic effects of estrogen. Moreover, our results from the transgenic animal model overexpressing MUL1 show that only males exhibit markers of cardiac hypertrophy, with females likely being protected by E2. This highlights MUL1 as a promising target for therapeutic interventions in cardiac diseases.

In conclusion, our study reveals a novel mechanism underlying the prevention of cardiac myocyte hypertrophy by 17-beta estradiol (E2). We found that NE induced mitochondrial network fragmentation, leading to reduced ATP levels and hypertrophy in cultured rat cardiomyocytes. Remarkably, pretreatment with E2 effectively prevented the detrimental effects induced by NE. Furthermore, we observed that E2 halted the NE-induced increases in both mRNA and protein levels of MUL1, a key regulator of mitochondrial dynamics. To further elucidate the role of MUL1 in this process, we employed MUL1 knockdown using small interfering RNA (siRNA), which effectively protected against NE-induced cardiomyocyte hypertrophy and mitochondrial dysfunction, mirroring the protective effects observed with E2 treatment. Conversely, overexpression of MUL1 using a MUL1 adenovirus negated the protective effects of E2 in terms of cardiomyocyte hypertrophy. This comprehensive analysis provides compelling evidence that E2 prevents cardiomyocyte hypertrophy by regulating MUL1, shedding light on a promising therapeutic target for cardiac diseases. Our findings offer new insights into the intricate interplay between estrogen signaling and mitochondrial dynamics in the context of cardiac health.

## Materials and methods

### Reagents

The antibodies used in this study were: anti-MUL1 (Cat. NBP1 59068), obtained from Novus Biologicals (Littleton, CO); Anti-pro atrial natriuretic peptide (ANP) (Cat. ab180649); anti-MFN2 (Cat. ab50838); anti-GPER30 (Cat. ab397242) from Abcam (Cambridge, UK); anti-DRP1-p616 (Cat. 3455S) from Cell Signaling Technologies (Danvers, MA); and anti-β-tubulin (Cat. T0198) from Sigma Aldrich (St. Louis, MO). NE (Cat. A7256), E2 (β-estradiol, 17-β-estradiol; Cat. E8875), pancreatin (Cat. P7545), and other chemicals were purchased from Sigma Aldrich (St. Louis, MO). Type 2 collagenase (Cat. 17101015) was from Thermo Fisher Scientific (Waltham, MA), and all the reagents used for protein electrophoresis were obtained from Biorad (Hercules, CA). The siRNAs targeting MUL1 transcripts were acquired from Integrated DNA Technologies (Coralville, IA), and their sequences were: siMUL1 5′-GGGAAAGUGUGUGCCUUAUTT-3′ (sense) and 3′-AUAAGGCACACACUUUCCCTT-5′ (antisense).

### NRVM culture and treatments

Cell cultures were maintained under standard cell culture conditions (humidified incubator, 37 °C, and 5% CO_2_). Neonatal Sprague Dawley male and female rats (1–3 day old) were euthanized by decapitation. Cardiomyocytes were isolated from rat hearts by enzymatic digestion using pancreatin (1.2 mg/mL) and collagenase (0.2 mg/mL) as described previously [18, 43]. Cells were pre-plated to discard non-myocyte cells and the myocyte-enriched fraction was plated at 1.0 × 10^6^ cells/mm^2^ on gelatin-precoated 35 mm dishes (Falcon, BD Biosciences, Oxford, UK) and grown in Dulbecco’s modified Eagle medium and M199 medium (DMEM/M199; ratio 4/1), with 10% (w/v) fetal bovine serum (FBS) for 24 h before the experiments. Cardiomyocyte cultures were at least 95% pure as evaluated by immunofluorescence using an anti-β-myosin heavy chain antibody (Vector Laboratories, Burlingame, CA, USA). Cells were grown and maintained at 37 °C in an incubator containing 95% O_2_ and 5% CO_2_. Following 24 h, the cells were stimulated with E2 (0, 10, 100 nM) for 6 h, followed by NE (20 μM) to induce cardiomyocyte hypertrophy. All experiments were conducted in the presence of 100 µM of BRDU to prevent the replication of fibroblasts, which may interfere with the results.

The knockdown experiments were conducted using Opti-MEM supplemented with MUL1-specific siRNAs. RNAiMAX from Invitrogen Thermo Fisher (Cat. 13778150) was used as a transfection reagent. The knockdown of MUL1 was confirmed by Western blotting and qPCR. Transfection with a human MUL1 (Applied Biological Materials Inc., abm, Cat. 31149054) and β-GAL adenovirus were performed using a multiplicity of infection (MOI) of 100 as we have previously described [18, 44]. After 24 h, the cells were incubated for 48 h with NE, either with or without E2.

### hiPSC Culture

hiPSCs derived from fibroblasts of a healthy individual (FiPS Ctrl2 SV4F1, registered in the National Stem Cell Bank, Carlos III Health Institute, Spain, and routinely tested for mycoplasma contamination) were utilized. The cells were cultured in mTeSR1 basal medium supplemented with 5× mTeSR1 supplement and 0.5% penicillin/streptomycin as an antibiotic. The cultures were maintained in 100 mm^2^ plates pre-coated with Matrigel. Cells were incubated at 37 °C in a humidified atmosphere with 5% CO_2_.

### Differentiation of hiPSCs into cardiomyocytes

hiPSCs were differentiated into cardiomyocytes following the protocol by Lian et al. [32]. hiPSCs maintained in mTeSR1 medium on Matrigel were dissociated into single cells using Accutase (Stemcell Technologies, Cat. 7922) for 5 min at 37 °C and seeded in Matrigel-coated 12-well plates at a density of 1.5 million cells/well, using mTeSR1 medium with 10 µM Y-27632 (Stemcell Technologies, Cat. 72304). Cells were cultured in mTeSR1 with daily medium changes for 3 days until confluence. Differentiation was initiated by treating the cells with 9 µM CHIR99021 (Sigma, Cat. SML1046) in RPMI medium supplemented with B27 without insulin, 1% GlutaMAX, 0.5% penicillin-streptomycin, 1% non-essential amino acids, and 0.1 mM 2-mercaptoethanol for 24 h (day 0). This was followed by treatment with 2 µM CHIR99021 for 48 h (days 1–2). On day 3, the medium was replaced with RPMI/B27 without insulin containing 5 µM IWP4 (TargetMol, Cat. T4245) and incubated for 48 h (days 3–4). From day 5, cells were cultured in RPMI/B27 without insulin for 48 h (days 5–6) and subsequently maintained in RPMI/B27 with insulin with medium changes every 2 days. Spontaneous beating was typically observed by day 8. On day 12, cells were purified with 10 mM lactate in RPMI/B27 without insulin for 48 h to eliminate non-cardiomyocytes. Cultures were maintained in a humidified atmosphere at 37 °C with 5% CO_2_ throughout the process.

### Induction of hypertrophy in CM-hiPSC

Once the cardiomyocytes were purified, they were incubated with 0.25% trypsin-EDTA (Gibco, Cat. 25200-056) for 5–8 minutes at 37 °C and seeded at a density of 1 million CM-hiPSC in 6-well plates. The cardiomyocytes were allowed to mature for 30 days and were maintained in RPMI medium supplemented with B27 with insulin (Gibco, Cat. 17504001), 1% GlutaMAX, 0.5% penicillin/streptomycin, 1% non-essential amino acids, and 0.1 mM 2-mercaptoethanol, with medium changes every 2 days. On day 30, 10 µM norepinephrine (Merck, Cat. A7256-1G) was added to the medium and maintained for 10 days with medium changes every 48 h.

### Real time qPCR

The real-time PCR experiments were performed using SYBR green from Thermo Fisher Scientific (Waltham, MA). The data from each transcript was normalized to 18S using the 2ΔΔCt method [44]. The rat primers used in this study were the following: MUL1 forward 5′-GGCCATTCTTTCAGAAGCAC-3′ and reverse 5′-TCCACAAACTGGCTGTTGAG-3′; regulator of calcineurin 1 exon 4 (RCAN 1.4) forward 5′-TCCTTGTCATATGTTCTGAAGAGGG-3′ and reverse 5′-CCCGTGAAAAAGCAGAATGC-3′; β-MHC forward 5′-AAGTCCTCCCTCAAGCTCCTAAGT-3′ and reverse 5′-TTGCTTTGCCTTTGCCC-3′; brain natriuretic peptide (BNP) forward 5′-TCCTTAATCTGTCGCCGCTG-3′ and reverse 5′-AGGCGCTGTCTTGAGACCTA-3′; ANP forward 5′-CTTCTTCCTCTTCCTGGCCT-3′ and reverse 5′-TTCATCGGTCTGCTCGCTCA-3′; and 18S forward 5′-CGGCTACCACATCCAAG-3′ and reverse 5′-CCAATGGATCCTCGTTA-3′.

The human primers used in this study were the following: MUL1 forward 5′-CCAAGAGACCGAGGAGATGC-3′ and reverse 5′-TGTTGTCCAGGACCAGTTCG-3′; brain natriuretic peptide (BNP) forward 5′-CTCCAGAGACATGGATCCCC-3′ and reverse 5′-GTTGCGCTGCTCCTGTAAC-3′; ANP forward 5′-CCGTGAGCTTCCTCCTTTTA-3′ and reverse 5′-CCAAATGGTCCAGCAAATTC-3’; and 18S forward 5′-CGGCTACCACATCCAAG-3′ and reverse 5′-CCAATGGATCCTCGTTA-3’.

The mice primers used in this study were the following: MUL1 forward 5’- GCAGGAACTCAAGGGAGCTAA-3’ and reverse 5′- CCACGAACTGGCTGTTGAGT-3′; ANP forward 5′- TCGTCTTGGCCTTTTGGC-3′ and reverse 5′- TCCAGGTGGTCTAGCAGGTTCT-3′; and 18S forward 5′- CCCTGCCCTTTGTACACACC-3′ and reverse 5′- CGATCCGAGGGCCTCACTA-3′.

### Western blot analysis

Once the designated treatment times were reached, the plates were washed three times with warm PBS. Cells were lysed with NP40 buffer supplemented with protease and phosphatase inhibitors. Homogenates were centrifuged at 21,600 × *g* for 3 min at 4 °C, and protein quantification was performed using the Bradford method (BioRad, Hercules, CA). The proteins were denatured in SDS buffer [18, 38, 44]. Equal amounts of protein were loaded and separated by molecular weight using SDS-PAGE electrophoresis. The polyacrylamide concentration was optimized based on the molecular weight of each target protein. The resolved protein samples were electrotransferred to PVDF membranes using a wet transfer system (400 mA for 90 min). The blocking agent was 5% non-fat milk dissolved in Tris-buffered saline containing 0.1% (v/v) Tween 20 (TBST). Membranes were incubated overnight with primary antibodies at 4 °C, followed by a 1 h incubation at room temperature with the respective peroxidase-linked secondary ATB. Chemiluminescence was detected with the Odyssey Fc Imaging System (LI-COR, Lincoln, NE) and densitometric analysis of the signals was carried out using the ImageJ software (NIH). Densitometric data were normalized to the values obtained for the loading control (β-tubulin).

### Mitochondrial morphology

Mitochondrial morphology was analyzed in coverslip-fixed NRVMs treated with anti-mtHSP70 (Abcam ab53098, 1:100) and visualized with an Alexa 488 secondary antibody, or in NRVMs incubated for 30 min with MTG-FM (400 nM) and maintained in Krebs solution (for live cell visualization) [44]. In fixed NRVMs, cell shape, and nuclei were visualized using rhodamine-phalloidin and DAPI, respectively. Confocal images were deconvolved with the ImageJ software (NIH), and then Z-stacks of thresholded images were volume-reconstituted using the VolumeJ plug-in. The number and individual volume of each object (mitochondria) were quantified using the ImageJ-3D Object Counter plug-in [18, 44].

### ATP measurements

As previously described [18, 29], the ATP content in cells was determined with a luciferin/luciferase-based assay (CellTiter-Glo Kit; Promega).

### In silico chromatin immunoprecipitation sequencing (ChIP-Seq) analysis

To investigate the potential regulation of MUL1 gene expression by androgen receptors (AR), estrogen receptor alpha (ERα), and estrogen-related receptors (ERRs), we conducted chromatin immunoprecipitation sequencing (ChIP-Seq) analysis. We sourced ChIP-Seq data from the ChIP-Atlas [45] and The Signaling Pathways Project [46] databases as of April 1st, 2023. We identified relevant ChIP-Seq studies by referencing the projects PRJNA135189, PRJNA193202, PRJNA227460, PRJNA235193, PRJNA235194, PRJNA264098, PRJNA305586, and PRJNA320640 in the above databases. Subsequently, we retrieved raw reads from these studies and matched them with the latest available genomes for *Homo sapiens* (T2T-CHM13 v2.0/hs1) and *Mus musculus* (GRCm39/mm39). To enhance the accuracy of our analyses, we recalculated the MACS2 (Model-based Analysis of ChIP-Seq) score for each identified binding site. Peaks with *p*-values lower than 1 × 10^−5^ were considered significant, indicating robustness in peak calling.

### Transgenic mouse model for MUL1 overexpression

The transgenic mouse model expressing MUL1 (strain name: C57BL/6J-Tg(aMHC-Mul1)) was generated by our collaborator Dr. Honglang Li (Institute of Model Animal, IMA, Wuhan University, China). Briefly, the Myh6 promoter was used to drive MUL1 gene expression. The transgene was designed with the following elements, from 5′ to 3′: a ~5.5 kbp mouse Myh6 minimal promoter sequence (which includes the 3′ exon of betaMHC, the first three exons of alphaMHC up to the start codon, and the intervening sequences) followed by a ~1059 bp cDNA encoding mitochondrial ubiquitin ligase activator of NFkappaB 1 (MUL1). These animals were initially generated on a C57BL/6 J background and subsequently backcrossed into the C57BL/6N background for at least nine generations. All experiments were performed on male and female mice aged 37 weeks with no randomization or blinding.

### Heart histology and WGA staining

Hearts were collected from all animals and processed for histological analysis. A transverse section of each heart was fixed in 10% (v/v) formalin prepared in 0.1 M phosphate buffer (pH 7.4) at room temperature for 24 h. After fixation, the samples were washed in 1× PBS for 1 h and dehydrated through a graded ethanol series (70%, 90%, and 100%) before being embedded in paraffin blocks. Four-micron thick sections were prepared and stained with Wheat Germ Agglutinin (WGA) CF 594 (Biotium, Cat. 29023-1) to assess the cross-sectional area of cardiomyocytes. To block nonspecific binding, the sections were incubated with 3% (w/v) Bovine Serum Albumin (BSA) in 1× PBS for 30 min, followed by incubation with WGA (1:500 dilution) for 1 h at room temperature in a humidified chamber. After staining, the sections were mounted using ProLong™ Gold Antifade Mountant with DAPI (Invitrogen, Cat. P36935) for fluorescence microscopy visualization. Images were captured at 20× magnification using the EVOS M5000 Imaging System. For analysis, all transverse heart sections were imaged and analyzed using ImageJ software (version 1.8.0) by converting images to 8-bit grayscale (Image > Type > 8-bit), applying a threshold (Image > Adjust > Threshold) to delineate cardiomyocyte boundaries, using the “Analyze Particles” tool with an area filter set to 20–1200 μm^2^ to select individual cardiomyocytes, and employing the “Multi-Measure” tool to calculate the cross-sectional area of each cardiomyocyte. Data were expressed as the mean cross-sectional area of cardiomyocytes for each experimental condition.

### Statistical analysis

Statistical analyses were performed using GraphPad Prism version 6 software. Data are presented as the mean ± SEM. Unless otherwise specified, each experiment included at least three independent biological replicates (*N*). For statistical comparisons, a two-sided Student’s *t*-test or one- or two-way ANOVA was used, as appropriate, followed by Tukey’s post-test. A *p*-value < 0.05 was considered statistically significant. For in vivo animal experiments, a two-sided Mann-Whitney nonparametric test was used for data analysis. No inclusion/exclusion criteria were used. No animals were excluded from the analysis.

Sample sizes for animal experiments were determined in accordance with international animal bioethics guidelines, applying the 3Rs principle (Replace, Reduce, Refine). The minimum number of animals required to achieve valid results was calculated based on the error associated with each technique. The sample size was determined using the formula *N* = 2 × (*Z*_α/2_ + *Z*_β_)^2^ × *s*^2^/*D*, as described by Taucher [47]. Here, *N* is the minimum number of observations required, *s* is the standard deviation of individual values (assumed equal across groups), *D* is the expected difference considered statistically significant, *Z*_α_ corresponds to a type I error probability of 5%, and *Z*_β_ corresponds to a type II error probability of 20%.

## Supplementary information

Supplemental Material

## Data Availability

Data will be made available on request.

## References

[1] Tanai E, Frantz S. Pathophysiology of heart failure. Compr Physiol. 2015;6:187–214. 26756631 10.1002/cphy.c140055

[2] Del Campo A, Perez G, Castro PF, Parra V, Verdejo HE. Mitochondrial function, dynamics and quality control in the pathophysiology of hfpef. Biochim Biophys Acta Mol Basis Dis. 2021;1867:166208 10.1016/j.bbadis.2021.166208 34214606 10.1016/j.bbadis.2021.166208

[3] Doherty DJ, Docherty KF, Gardner RS. Review of the national institute for health and care excellence guidelines on chronic heart failure. Heart. 2024;110:466–75. 10.1136/heartjnl-2022-322164 38191272 10.1136/heartjnl-2022-322164

[4] Bazgir F, Nau J, Nakhaei-Rad S, Amin E, Wolf MJ, Saucerman JJ, et al. The microenvironment of the pathogenesis of cardiac hypertrophy. Cells. 2023;12:1780. 10.3390/cells12131780 37443814 10.3390/cells12131780 PMC10341218

[5] Ritterhoff J, Tian R. Metabolic mechanisms in physiological and pathological cardiac hypertrophy: new paradigms and challenges. Nat Rev Cardiol. 2023;20:812–29. 10.1038/s41569-023-00887-x 37237146 10.1038/s41569-023-00887-x

[6] Nakamura M, Sadoshima J. Mechanisms of physiological and pathological cardiac hypertrophy. Nat Rev Cardiol. 2018;15:387–407. 10.1038/s41569-018-0007-y 29674714 10.1038/s41569-018-0007-y

[7] Wu J, Dai F, Li C, Zou Y. Gender differences in cardiac hypertrophy. J Cardiovasc Transl Res. 2020;13:73–84. 10.1007/s12265-019-09907-z 31418109 10.1007/s12265-019-09907-z

[8] Agabiti-Rosei E, Muiesan ML. Left ventricular hypertrophy and heart failure in women. J Hypertens Suppl. 2002;20:S34–8. 12183849

[9] Knowlton AA, Lee AR. Estrogen and the cardiovascular system. Pharmacol Ther. 2012;135:54–70. 10.1016/j.pharmthera.2012.03.007 22484805 10.1016/j.pharmthera.2012.03.007 PMC5688223

[10] Subramanya V, Zhao D, Ouyang P, Lima JA, Vaidya D, Ndumele CE, et al. Sex hormone levels and change in left ventricular structure among men and post-menopausal women: the multi-ethnic study of atherosclerosis (mesa). Maturitas. 2018;108:37–44. 10.1016/j.maturitas.2017.11.006 29290213 10.1016/j.maturitas.2017.11.006 PMC5752123

[11] Miya Y, Sumino H, Ichikawa S, Nakamura T, Kanda T, Kumakura H, et al. Effects of hormone replacement therapy on left ventricular hypertrophy and growth-promoting factors in hypertensive postmenopausal women. Hypertens Res. 2002;25:153–9. 10.1291/hypres.25.153 12047028 10.1291/hypres.25.153

[12] Babiker FA, De Windt LJ, van Eickels M, Grohe C, Meyer R, Doevendans PA. Estrogenic hormone action in the heart: regulatory network and function. Cardiovasc Res. 2002;53:709–19. 10.1016/s0008-6363(01)00526-0 11861041 10.1016/s0008-6363(01)00526-0

[13] Osborne CK, Zhao H, Fuqua SA. Selective estrogen receptor modulators: structure, function, and clinical use. J Clin Oncol. 2000;18:3172–86. 10.1200/JCO.2000.18.17.3172 10963646 10.1200/JCO.2000.18.17.3172

[14] Russell JK, Jones CK, Newhouse PA. The role of estrogen in brain and cognitive aging. Neurotherapeutics. 2019;16:649–65. 10.1007/s13311-019-00766-9 31364065 10.1007/s13311-019-00766-9 PMC6694379

[15] Faltas CL, LeBron KA, Holz MK. Unconventional estrogen signaling in health and disease. Endocrinology. 2020;161:bqaa030 10.1210/endocr/bqaa030 32128594 10.1210/endocr/bqaa030 PMC7101056

[16] Guajardo-Correa E, Silva-Aguero JF, Calle X, Chiong M, Henriquez M, Garcia-Rivas G, et al. Estrogen signaling as a bridge between the nucleus and mitochondria in cardiovascular diseases. Front Cell Dev Biol. 2022;10:968373. 10.3389/fcell.2022.968373 36187489 10.3389/fcell.2022.968373 PMC9516331

[17] Rattanasopa C, Phungphong S, Wattanapermpool J, Bupha-Intr T. Significant role of estrogen in maintaining cardiac mitochondrial functions. J Steroid Biochem Mol Biol. 2015;147:1–9. 10.1016/j.jsbmb.2014.11.009 25448746 10.1016/j.jsbmb.2014.11.009

[18] Pennanen C, Parra V, Lopez-Crisosto C, Morales PE, Del Campo A, Gutierrez T, et al. Mitochondrial fission is required for cardiomyocyte hypertrophy mediated by a ca2+-calcineurin signaling pathway. J Cell Sci. 2014;127:2659–71. 10.1242/jcs.139394 24777478 10.1242/jcs.139394 PMC4058110

[19] Morales PE, Arias-Duran C, Avalos-Guajardo Y, Aedo G, Verdejo HE, Parra V, et al. Emerging role of mitophagy in cardiovascular physiology and pathology. Mol Aspects Med. 2020;71:100822. 10.1016/j.mam.2019.09.006 31587811 10.1016/j.mam.2019.09.006

[20] Sotomayor-Flores C, Rivera-Mejias P, Vasquez-Trincado C, Lopez-Crisosto C, Morales PE, Pennanen C, et al. Angiotensin-(1-9) prevents cardiomyocyte hypertrophy by controlling mitochondrial dynamics via mir-129-3p/pkia pathway. Cell Death Differ. 2020;27:2586–604. 10.1038/s41418-020-0522-3 32152556 10.1038/s41418-020-0522-3 PMC7429871

[21] Neuspiel M, Schauss AC, Braschi E, Zunino R, Rippstein P, Rachubinski RA, et al. Cargo-selected transport from the mitochondria to peroxisomes is mediated by vesicular carriers. Curr Biol. 2008;18:102–8. 10.1016/j.cub.2007.12.038 18207745 10.1016/j.cub.2007.12.038

[22] Peng J, Ren KD, Yang J, Luo XJ. Mitochondrial e3 ubiquitin ligase 1: a key enzyme in regulation of mitochondrial dynamics and functions. Mitochondrion. 2016;28:49–53. 10.1016/j.mito.2016.03.007 27034206 10.1016/j.mito.2016.03.007

[23] Braschi E, Zunino R, McBride HM. Mapl is a new mitochondrial sumo e3 ligase that regulates mitochondrial fission. EMBO Rep. 2009;10:748–54. 10.1038/embor.2009.86 19407830 10.1038/embor.2009.86 PMC2727426

[24] Bae S, Kim SY, Jung JH, Yoon Y, Cha HJ, Lee H, et al. Akt is negatively regulated by the mulan e3 ligase. Cell Res. 2012;22:873–85. 10.1038/cr.2012.38 22410793 10.1038/cr.2012.38 PMC3343661

[25] Attaix D, Taillandier D. The missing link: Mul1 signals mitophagy and muscle wasting. Cell Metab. 2012;16:551–2. 10.1016/j.cmet.2012.10.013 23140636 10.1016/j.cmet.2012.10.013

[26] Prudent J, Zunino R, Sugiura A, Mattie S, Shore GC, McBride HM. Mapl sumoylation of drp1 stabilizes an er/mitochondrial platform required for cell death. Mol Cell. 2015;59:941–55. 10.1016/j.molcel.2015.08.001 26384664 10.1016/j.molcel.2015.08.001

[27] Zhao Y, Ponnusamy M, Liu C, Tian J, Dong Y, Gao J, et al. Mir-485-5p modulates mitochondrial fission through targeting mitochondrial anchored protein ligase in cardiac hypertrophy. Biochim Biophys Acta Mol Basis Dis. 2017;1863:2871–81. 10.1016/j.bbadis.2017.07.034 28782654 10.1016/j.bbadis.2017.07.034

[28] Calle X, Garrido-Moreno V, Lopez-Gallardo E, Norambuena-Soto I, Martinez D, Penaloza-Otarola A, et al. Mitochondrial e3 ubiquitin ligase 1 (mul1) as a novel therapeutic target for diseases associated with mitochondrial dysfunction,. IUBMB Life. 2022;74:850–65. 10.1002/iub.2657 35638168 10.1002/iub.2657

[29] Parra V, Altamirano F, Hernandez-Fuentes CP, Tong D, Kyrychenko V, Rotter D, et al. Down syndrome critical region 1 gene, rcan1, helps maintain a more fused mitochondrial network. Circ Res. 2018;122:e20–e33. 10.1161/CIRCRESAHA.117.311522 29362227 10.1161/CIRCRESAHA.117.311522 PMC5924463

[30] Vasquez-Trincado C, Garcia-Carvajal I, Pennanen C, Parra V, Hill JA, Rothermel BA, et al. Mitochondrial dynamics, mitophagy and cardiovascular disease. J Physiol. 2016;594:509–25. 10.1113/JP271301 26537557 10.1113/JP271301 PMC5341713

[31] Mehta J, Kling JM, Manson JE. Risks, benefits, and treatment modalities of menopausal hormone therapy: Current concepts. Front Endocrinol (Lausanne). 2021;12:564781. 10.3389/fendo.2021.564781 33841322 10.3389/fendo.2021.564781 PMC8034540

[32] Lian X, Zhang J, Azarin SM, Zhu K, Hazeltine LB, Bao X, et al. Directed cardiomyocyte differentiation from human pluripotent stem cells by modulating wnt/beta-catenin signaling under fully defined conditions. Nat Protoc. 2013;8:162–75. 10.1038/nprot.2012.150 23257984 10.1038/nprot.2012.150 PMC3612968

[33] Pedram A, Razandi M, Aitkenhead M, Levin ER. Estrogen inhibits cardiomyocyte hypertrophy in vitro. Antagonism of calcineurin-related hypertrophy through induction of mcip1. J Biol Chem. 2005;280:26339–48. 10.1074/jbc.M414409200 15899894 10.1074/jbc.M414409200 PMC1249515

[34] Pei H, Wang W, Zhao D, Su H, Su G, Zhao Z. G protein-coupled estrogen receptor 1 inhibits angiotensin ii-induced cardiomyocyte hypertrophy via the regulation of pi3k-akt-mtor signalling and autophagy. Int J Biol Sci. 2019;15:81–92. 10.7150/ijbs.28304 30662349 10.7150/ijbs.28304 PMC6329915

[35] Goncalves GK, Scalzo S, Alves AP, Agero U, Guatimosim S, Reis AM. Neonatal cardiomyocyte hypertrophy induced by endothelin-1 is blocked by estradiol acting on gper. Am J Physiol Cell Physiol. 2018;314:C310–C322. 10.1152/ajpcell.00060.2017 29167148 10.1152/ajpcell.00060.2017

[36] Hoa N, Ge L, Korach KS, Levin ER. Estrogen receptor beta maintains expression of klf15 to prevent cardiac myocyte hypertrophy in female rodents. Mol Cell Endocrinol. 2018;470:240–50. 10.1016/j.mce.2017.11.004 29127073 10.1016/j.mce.2017.11.004 PMC6242344

[37] Chen P, Li B, Ou-Yang L. Role of estrogen receptors in health and disease. Front Endocrinol (Lausanne). 2022;13:839005 10.3389/fendo.2022.839005 36060947 10.3389/fendo.2022.839005 PMC9433670

[38] Vasquez-Trincado C, Navarro-Marquez M, Morales PE, Westermeier F, Chiong M, Parra V, et al. Myristate induces mitochondrial fragmentation and cardiomyocyte hypertrophy through mitochondrial e3 ubiquitin ligase mul1. Front Cell Dev Biol. 2023;11:1072315. 10.3389/fcell.2023.1072315 37051468 10.3389/fcell.2023.1072315 PMC10083258

[39] Fang L, Moore XL, Gao XM, Dart AM, Lim YL, Du XJ. Down-regulation of mitofusin-2 expression in cardiac hypertrophy in vitro and in vivo. Life Sci. 2007;80:2154–60. 10.1016/j.lfs.2007.04.003 17499311 10.1016/j.lfs.2007.04.003

[40] Yu H, Guo Y, Mi L, Wang X, Li L, Gao W. Mitofusin 2 inhibits angiotensin ii-induced myocardial hypertrophy. J Cardiovasc Pharmacol Ther. 2011;16:205–11. 10.1177/1074248410385683 21106870 10.1177/1074248410385683

[41] Maas A. Hormone therapy and cardiovascular disease: benefits and harms. Best Pract Res Clin Endocrinol Metab. 2021;35:101576 10.1016/j.beem.2021.101576 34556415 10.1016/j.beem.2021.101576

[42] Shufelt CL, Manson JE. Menopausal hormone therapy and cardiovascular disease: the role of formulation, dose, and route of delivery. J Clin Endocrinol Metab. 2021;106:1245–54. 10.1210/clinem/dgab042 33506261 10.1210/clinem/dgab042 PMC8063246

[43] Galvez A, Morales MP, Eltit JM, Ocaranza P, Carrasco L, Campos X, et al. A rapid and strong apoptotic process is triggered by hyperosmotic stress in cultured rat cardiac myocytes. Cell Tissue Res. 2001;304:279–85. 10.1007/s004410100358 11396721 10.1007/s004410100358

[44] Parra V, Verdejo HE, Iglewski M, Del Campo A, Troncoso R, Jones D, et al. Insulin stimulates mitochondrial fusion and function in cardiomyocytes via the akt-mtor-nfkappab-opa-1 signaling pathway. Diabetes. 2014;63:75–88. 10.2337/db13-0340 24009260 10.2337/db13-0340 PMC3868041

[45] Zou Z, Ohta T, Miura F, Oki S. Chip-atlas 2021 update: a data-mining suite for exploring epigenomic landscapes by fully integrating chip-seq, atac-seq and bisulfite-seq data. Nucleic Acids Res. 2022;50:W175–W182. 10.1093/nar/gkac199 35325188 10.1093/nar/gkac199 PMC9252733

[46] Ochsner SA, Abraham D, Martin K, Ding W, McOwiti A, Kankanamge W, et al. The signaling pathways project, an integrated ‘omics knowledgebase for mammalian cellular signaling pathways. Sci Data. 2019;6:252. 10.1038/s41597-019-0193-4 31672983 10.1038/s41597-019-0193-4 PMC6823428

[47] E Taucher, Bioestadistica, Primera ed., Editorial Universitaria (1997).

