# Supplementary material for: 17-beta estradiol prevents cardiac myocyte hypertrophy by regulating mitochondrial E3 ubiquitin ligase 1
Source: Cell Death Dis. 2025 Feb 19;16(1):111. doi: 10.1038/s41419-025-07389-3 (PMC11839923; doi:10.1038/s41419-025-07389-3)

SUPPLEMENTARY FIGURE LEGENDS

Suppl. Fig. 1. Effect of E2 on cardiomyocyte hypertrophy. Cultured cardiomyocytes were pretreated with E2 (0, 1, 10, 100 nM) for 48 h. **(A)** Protein extracts were obtained. ANP protein levels were determined by western blotting (N=5). Β-tubulin was used as a loading control. **(B)** BNP (N=4), **(C)** ANP (N=6), **(D)** RCAN1.4 (N=7), and **(E)** β-MHC (N=3) mRNA levels were assessed by RT-qPCR. 18S RNA was used to normalize the data. Values correspond to the mean ± SEM. Each independent experiment is displayed as a dot in the graphs. Results were analyzed using a one-way ANOVA followed by multiple Tukey's comparisons. No statistical difference was found.

Suppl. Fig. 2: Effect of E2 on cardiomyocyte hypertrophy and mitochondrial morphology. Primary cultures of NRVM seeded on coverslips were treated with E2 (0, 1, 10, 100 nM) for 48 h. **(A)** Cardiomyocytes were fixed, mitochondria were detected by immunolabeling with mtHsp70 and using a secondary antibody labeled with Alexa 488 (green), and cell shape was visualized by staining with rhodamine-phalloidin (red) and nuclei with DAPI (blue). Images of cardiomyocytes were captured by confocal microscopy. **(A)** Representative confocal microscopy images. **(B)** Cell area (N=4) and cell perimeter (N=3). **(C)** Relative mitochondrial number (N=4). **(D)** Relative mitochondrial volume (N=4). Values correspond to the mean ± SEM. Each independent experiment is displayed as a dot in the graphs. Results were analyzed using one-way ANOVA followed by multiple Tukey's comparisons. No statistical difference was found.

Suppl. Fig. 3: Effect of E2 on mitochondrial dynamic proteins in normal and NE-dependent hypertrophic cardiomyocytes. Primary cultured NRVMs were treated with E2 (0, 1, 10, 100 nM) for 6 h and then with **(A, C)** or without **(B, D)** NE (20 µM) for 48 h. **(A)** Protein extracts were obtained and analyzed by western blotting using anti Mfn2 (N=4) **(A, B)** and phospho Ser^{616} DRP-1 (p-DRP1 Ser^{616}) (N=4-5) **(C, D)** antibodies. β-tubulin was used as a loading control. The data correspond to the mean ± SEM. Each independent experiment is displayed as a dot in the graphs. Results were analyzed using one-way ANOVA followed by multiple Tukey's comparisons. $^{*}p<0.05$.

Suppl. Fig. 4: Uncropped Western blots. The uncropped Western blots for all the figures are presented here in the order of their corresponding figures and panels.

Supplementary Figure 1

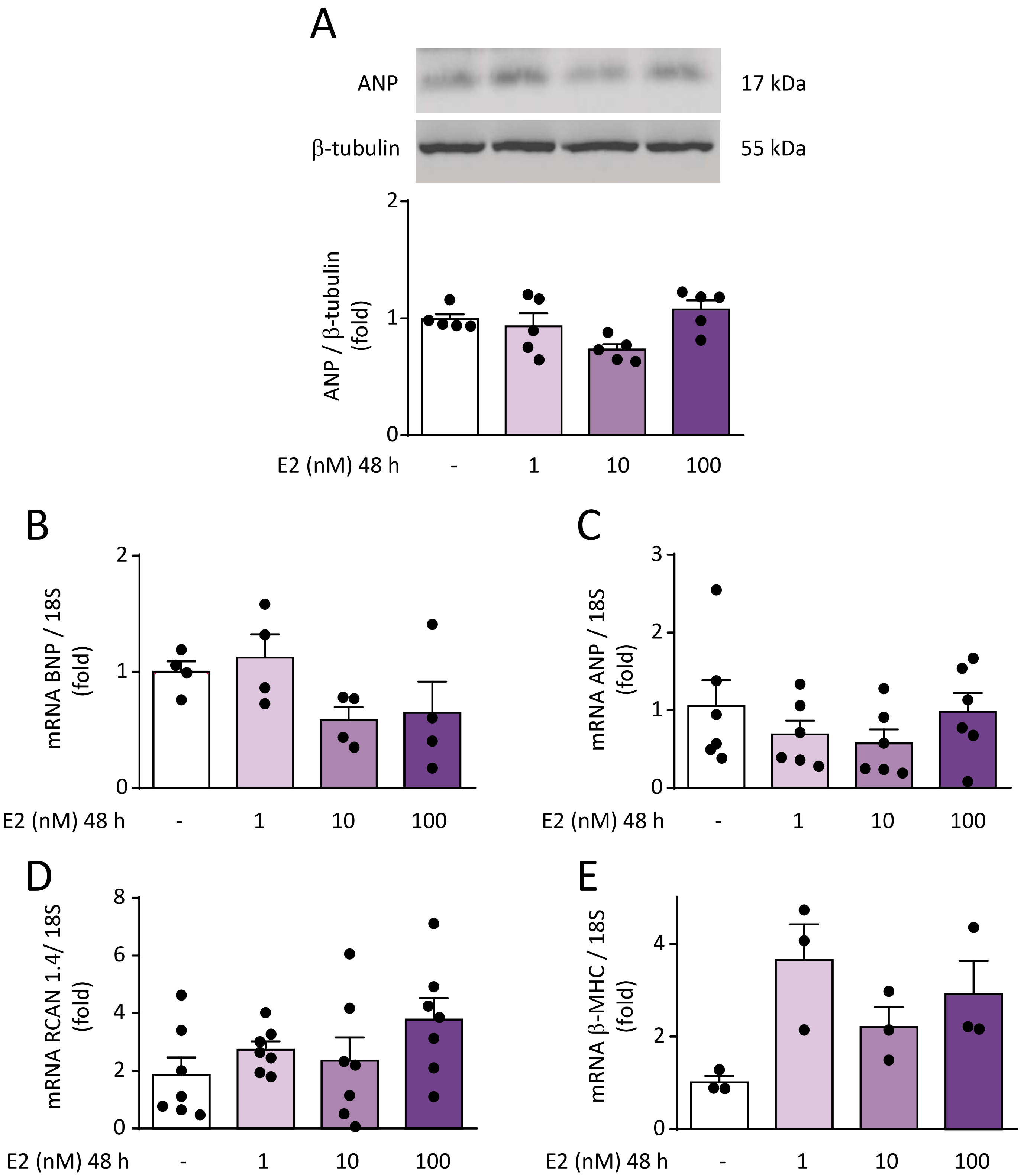

Supplementary Figure 2

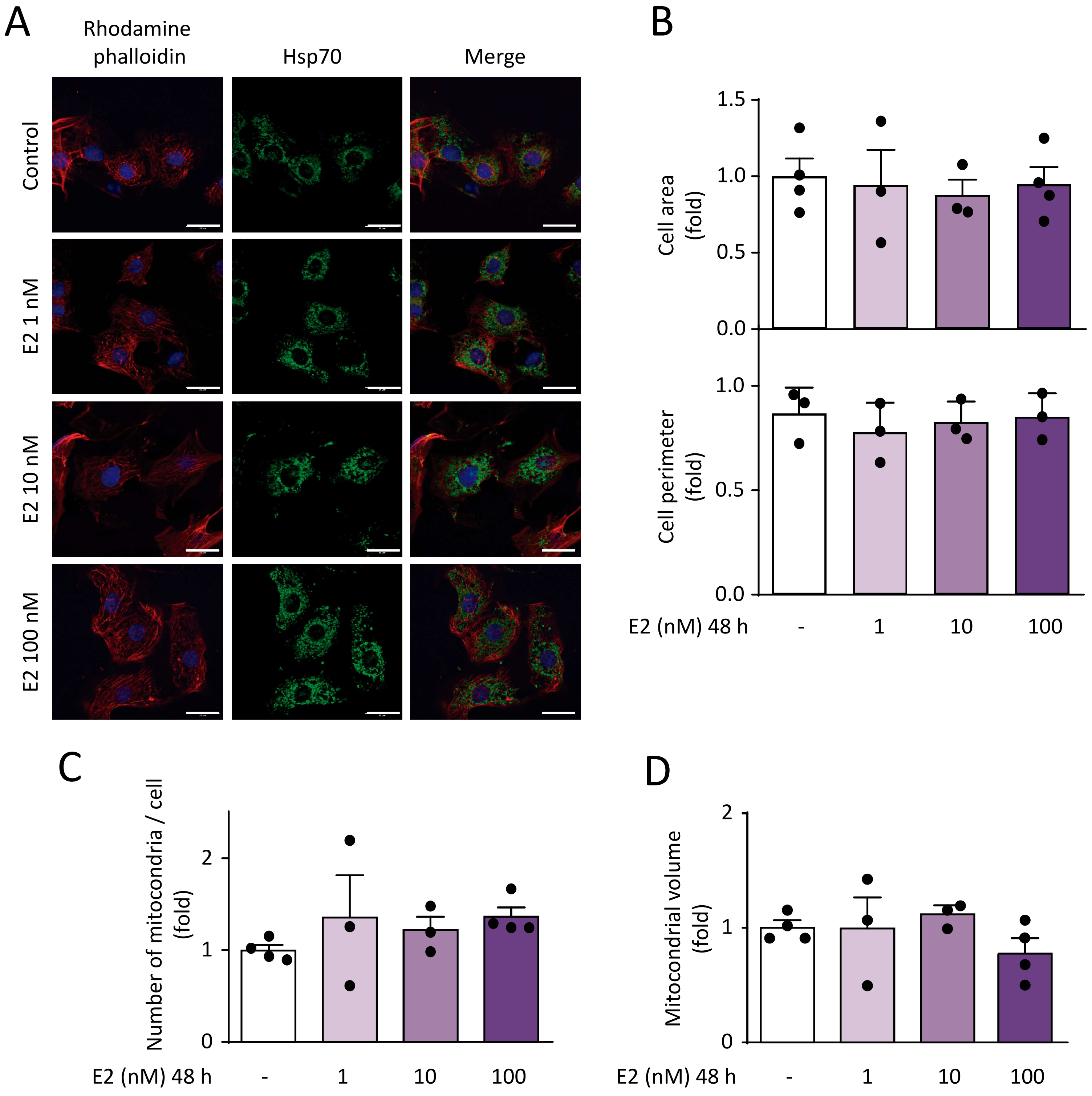

Supplementary Figure 3

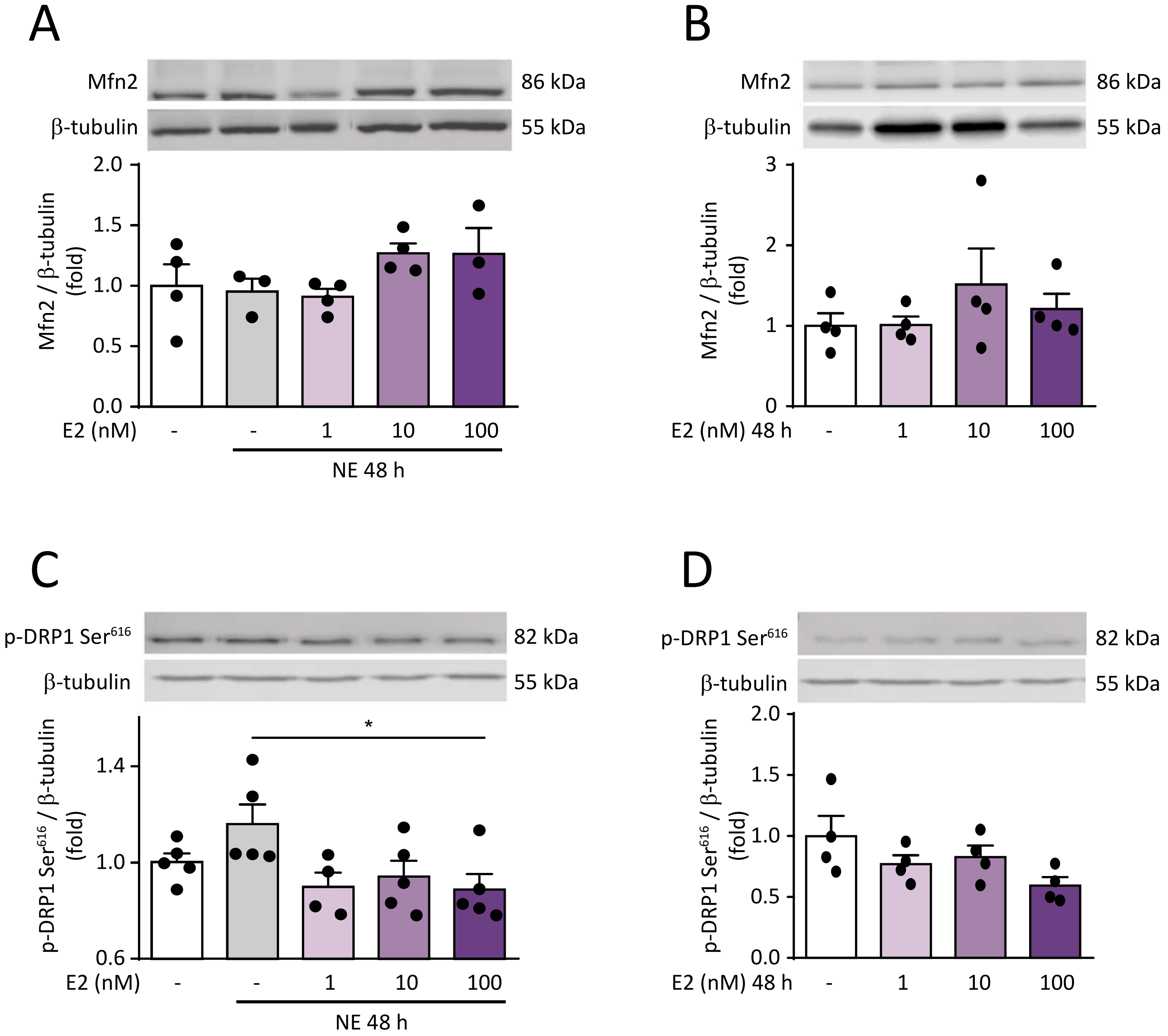

Supplementary Figure 4

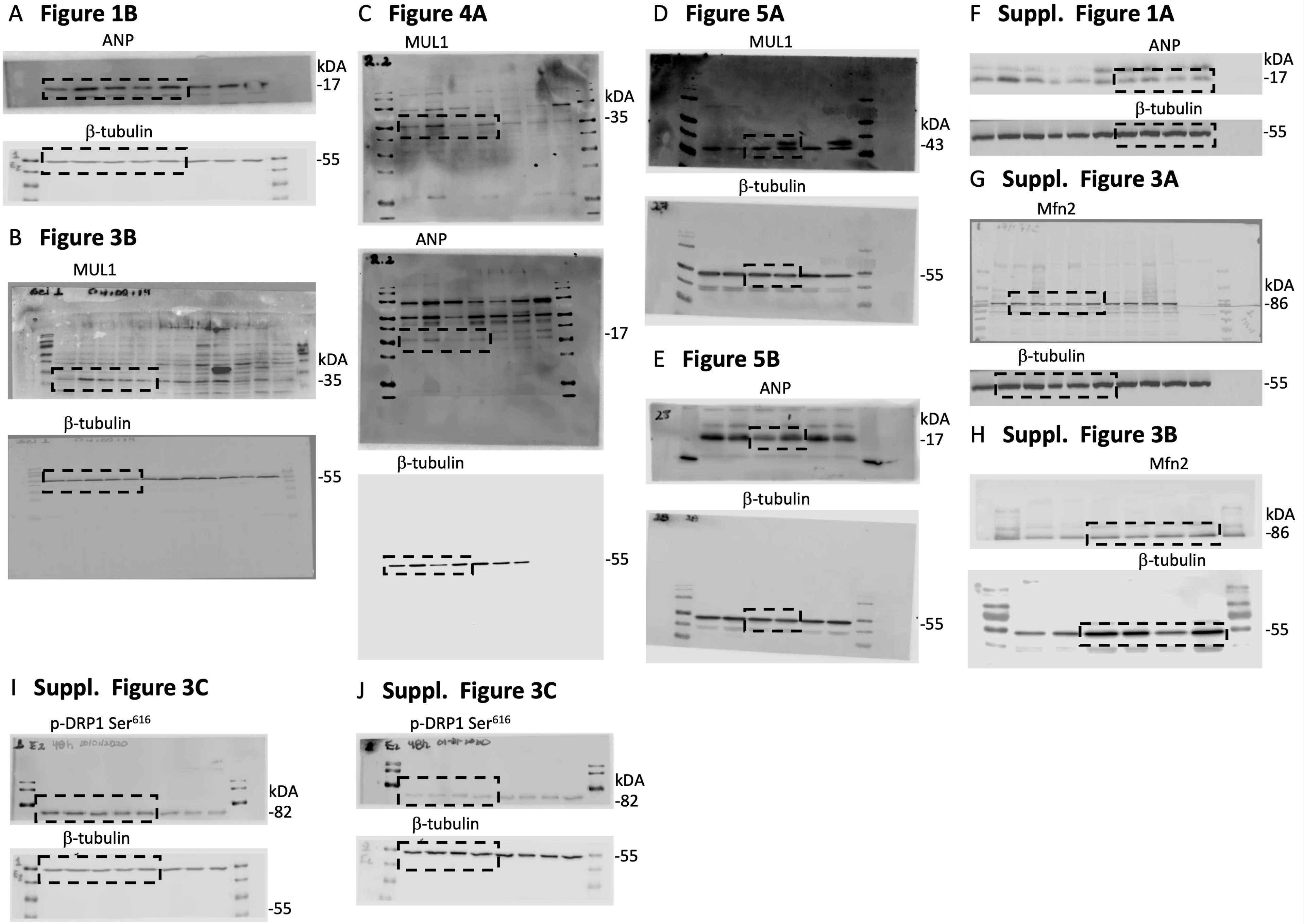

Supplement: Supplementary file 1 — Supplemental Material [file 41419_2025_7389_MOESM1_ESM.pdf]
